# MicroRNA-122 regulates caspase-8 and promotes the apoptosis of mouse cardiomyocytes

**DOI:** 10.1590/1414-431X20165760

**Published:** 2017-02-06

**Authors:** Z.W. Zhang, H. Li, S.S. Chen, Y. Li, Z.Y. Cui, J. Ma

**Affiliations:** Department of Cardiology, Jining No.1 People’s Hospital, Jining, Shandong Province, China

**Keywords:** Cardiomyocyte, Apoptosis, miR-122, Caspase-8, Myocardial infarction

## Abstract

Cardiomyocyte apoptosis plays key roles in the pathogenesis of heart diseases such as myocardial infarction. MicroRNAs are important regulators of gene expression, which are also involved in the regulation of cardiomyocyte apoptosis. However, cardiomyocyte apoptosis regulated by microRNA (miR)-122 is largely unexplored. The aim of this study focused on the role of miR-122 in cardiomyocyte apoptosis. Cardiomyocytes were isolated from neonatal mice and primarily cultured. MiR-122 mimic and inhibitor were transfected to cardiomyocytes and verified by qRT-PCR. Cell viability and apoptosis post-transfection were assessed by MTT assay and flow cytometry, respectively. Changes in expression of caspase-8 were quantified by qRT-PCR and western blot. Results showed that miR-122 mimic and inhibitor successfully induced changes in miR-122 levels in cultured cardiomyocytes (P<0.01). MiR-122 overexpression suppressed viability and promoted apoptosis of cardiomyocytes (P<0.05), and miR-122 knockdown promoted cell viability and inhibited apoptosis (P<0.05). The mRNA and protein levels of caspase-8 were elevated by miR-122 overexpression (P<0.01) and reduced by miR-122 knockdown (P<0.001). These results suggest an inductive role of miR-122 in cardiomyocyte apoptosis, which may be related to its regulation on caspase-8.

## Introduction

Cardiomyocyte apoptosis is closely associated with the pathogenesis of many heart diseases, including myocardial infarction, cardiac hypertrophy and heart failure (1,2). Myocardial infarction, for example, is attributed to the myocardial necrosis induced by acute or persistent hypoxia-ischemia. It is usually life-threatening, affecting an increasing number of lives year after year, especially in developed areas (3). In the initial stage of myocardial infarction, cardiomyocytes on the coronary artery are impaired by hypoxia-ischemia and go through cell death, which plays key roles in the tissue damage accompanying myocardial infarction (4). Studies on molecular mechanisms have shed light on the involvement of multiple apoptotic factors, such as the caspase family, in the regulation of cardiomyocyte apoptosis during myocardial infarction (5).

MicroRNAs (miRNAs) are small non-coding RNAs that can bind to specific target sequences of mRNAs to regulate gene expression post-transcriptionally, and have become a prevalent topic due to their critical influence on the pathogenesis of diseases including heart diseases (6,7). Circulating miRNAs like miR-1, miR-133a and miR-499 are promising biomarkers for the diagnosis of acute myocardial infarction (8,9). Restrained miR-24 inhibits endothelial apoptosis and reduces myocardial infarct size (10). Knockdown of miR-208a helps to attenuate apoptosis and improve cardiac function after myocardial infarction (11). Thus, miRNA-based therapeutics have a potential role in the treatment of heart diseases (12).

Multiple studies have documented that miR-122 can regulate hepatitis C and hepatocellular carcinoma. MiR-122 stimulated the translation of hepatitis C virus RNA, and thus serves as a potential target for treating hepatitis C (13). In hepatocellular carcinoma, miR-122 can induce cell apoptosis to suppress tumor progression (14). However, to date, miR-122 has not been investigated as a modulator in cardiomyocyte apoptosis. This study aimed to uncover the impact of miR-122 on cardiomyocyte apoptosis, help understand the role of miR-122 on cardiomyocyte apoptosis, and provide potential targets for treating myocardial infarction and other heart diseases.

## Material and Methods

### Cell culture

This study was performed according to the National Institutes of Health’s Guide for the Care and Use of Laboratory Animals and was approved by the Ethics Committee of Jining No.1 People’s Hospital (Jining, China). Mouse cardiomyocytes were isolated and cultured based on a previous report (15). Neonatal BALB/c mice (20 individuals, 3 days old, Vital River Laboratories, China) were anesthetized with methoxyflurane (1.5%, Hebei Institute of Medicine, Shijiazhuang, China) and immersed in 75% ethyl alcohol. The thoracic cavity was opened and the heart was sampled. The cardiac muscle tissue was isolated, washed in phosphate-buffered saline (PBS) for 3 times and then cut into pieces of about 1 mm^3^. Tissue was digested in 0.125% Trypsin (Sigma-Aldrich, China) for 3 min at 37°C, and the supernatant was discarded, after which the tissue was digested in 0.1% collagenase I (Sigma-Aldrich) for 7 min at 37°C. Digestion was terminated by Dulbecco’s minimum essential medium (DMEM, Gibco, USA) with 15% fetal bovine serum (FBS, Gibco). The supernatant was collected and centrifuged at 179 *g* for 8 min at 4°C. Cells were resuspended in DMEM with 15% FBS and cultured in humid air with 5% CO_2_ at 37°C. After 60 min of incubation, cells in the supernatant were isolated for further culture, while adherent cells were discarded. During the first 2 days of cell culture, 5-bromo-2′-deoxyuridine (100 μM, Sigma-Aldrich) was added to the medium to inhibit the proliferation of non-cardiomyocytes. The medium was changed every other day.

### Cell transfection

After 2 days of primary culture, the mouse cardiomyocytes were collected for cell transfection. Single-cell suspension was prepared and seeded on 12-well plates (5×10^4^ cells/well) to reach a confluency of about 90%. miR-122 mimic (50 nM), miR-122 inhibitor (100 nM) or the scrambled sequence (100 nM), which served as negative control (NC) (RiboBio, China) were transfected to the cells in antibiotic- and serum-free medium using Lipofectamine 2000 (Invitrogen, USA), according to the manufacturer’s instructions. Cells without transfection were used as the Control group. At 48-h post-transfection, miR-122 level was quantified by qRT-PCR to evaluate the effect of transfection.

### Cell viability assay

At 0, 24, 48, and 72 h post-transfection, cell viability was assessed by the MTT method using MTT Cell Proliferation Assay Kit (ATCC, USA) according to the manufacturer’s instructions. Briefly, 1×10^4^ cells were plated in 96-well plates and 10 μL MTT Reagent was added. The plates were incubated at 37°C for 4 h, and then 100 μL detergent reagent was added to each well and the plates were kept in the dark at room temperature for 2 h. The optical density was measured at 570 nm using a microplate reader TECAN GENios Pro (Tecan, Switzerland).

### Cell apoptosis assay

At 48-h post-transfection, cell apoptosis was measured by flow cytometry after cells were stained with fluorescein isothiocyanate (FITC) and propidium iodide (PI) using Annexin V-FITC Apoptosis Kit (BioVision, USA) according to the manufacturer’s instructions. Briefly, 1×10^5^ cells were washed in cold PBS twice and resuspended in 100 μL binding buffer. Annexin V-FITC (2 μL) was added, and the cells were incubated on ice in the dark for 15 min. PBS (400 μL) and 1 μL PI were added, and detection was performed on a flow cytometer Attune NxT (Invitrogen). The FITC-positive and PI-negative cells were considered apoptotic cells.

### Western blot

Protein samples from transfected cells were extracted using ProteoPrep Total Extraction Sample Kit (Sigma-Aldrich) at 48-h post-transfection and quantified by Bio-Rad Protein Assay (Bio-Rad, USA). Proteins were analyzed by sodium dodecyl sulfate-polyacrylamide gel electrophoresis and then transferred to a nitrocellulose membrane (Invitrogen). The membrane was blocked in 5% skim milk (in PBS) for 4 h at 4°C and incubated in the rabbit polyclonal specific antibodies for caspase-8 (1:1000, ab25901, Abcam, UK) overnight at 4°C. GAPDH antibodies (ab9485) were used as an internal reference. After being washed in PBS 5 times, the membrane was incubated in goat anti-rabbit IgG H&L (horseradish peroxidase-conjugated, 1:2000, ab97051) for 1 h at room temperature and washed in PBS again 5 times. Signals were visualized by ECL Plus Western Blotting Substrate (Piece, USA) and quantified by ImageJ 1.49 (National Institutes of Health, USA).

### qRT-PCR

Small RNA from transfected cells was extracted using mirVana miRNA Isolation Kit (Ambion, USA) according to the manufacturer’s instructions at 48-h post-transfection. Reverse transcription was performed using the specific primer for mmu-miR-122-5p (5′-CTCAA CTGGT GTCGT GGAGT CGGCA ATTCA GTTGA GCAAA CACC-3′) and SuperScript III Reverse Transcriptase (Invitrogen). qRT-PCR was carried out on LightCycler 480 (Roche, Switzerland) using SYBR Green I Master (Roche) and the specific primers amplifying miR-122 (forward: 5′-ACACT CCAGC TGGGT GGAGT GTGAC AAT-3′ and reverse: 5′-TGGTG TCGTG GAGTC G-3′). Data were calculated by the 2^-ΔΔCt^ method (16) and normalized by *U6* (forward: 5′-GCATG ACGTC TGCTT TGGA-3′ and reverse: 5′-CCACA ATCAT TCTGC CATCA-3′).

The same procedure was performed to quantify mRNA level of caspase-8 from total RNA extracted with TRIzol (Invitrogen). The specific primers for detecting caspase-8 mRNA in qRT-PCR were: forward 5′-ACCGA GATCC TGTGA ATGGA ACC-3′ and reverse 5′-TAAGA ATGTC ATCTC CTTGA GGA-3′. Data were normalized to *Gapdh* (forward: 5′-TCAAC AGCAA CTCCC ACTCT TCCA-3′ and reverse: 5′-ACCCT GTTGC TGTAG CCGTA TTCA-3′).

### Statistical analysis

Data were from 5 independent experiments done in triplicate. Data are reported as means±SD and analyzed using Student’s *t*-test in SPSS 20 (IBM, USA). P<0.05 was considered to be statistically significant.

## Results

### MiR-122 inhibited viability and promoted apoptosis of mouse cardiomyocytes

**Figure 1:**
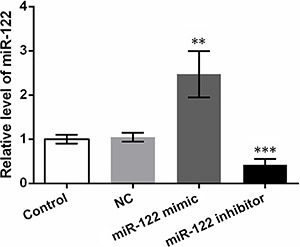
Levels of miR-122 in transfected mouse cardiomyocytes. Mouse cardiomyocytes were transfected with miR-122 mimic, miR-122 inhibitor or the negative control (NC). qRT-PCR was performed at 48-h post-transfection to quantify miR-122. Control: untransfected cells. Data are reported as means±SD. **P<0.01, ***P<0.001 compared to NC group (t-test).

MiR-122 mimic or inhibitor was transfected to the primarily cultured mouse cardiomyocytes to up-regulate or down-regulate the expression of miR-122, respectively. Transfection with NC did not significantly change miR-122 level compared to the Control group (Figure 1), but miR-122 mimic or inhibitor significantly elevated or knocked down miR-122 levels (P<0.01 and P<0.001), confirming the effective cell transfection. Thus, the transfected cells were used in the following detection.

**Figure 2:**
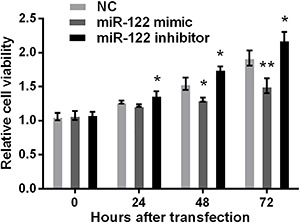
Viability of transfected mouse cardiomyocytes. Mouse cardiomyocytes were transfected with miR-122 mimic, miR-122 inhibitor or the negative control (NC). MTT assay was performed at 0, 24, 48, and 72 h post-transfection and absorbance at 570 nm was measured. Data are reported as means±SD. *P<0.05, **P<0.01 compared to NC group (*t*-test).

MTT assay indicated that the cardiomyocytes transfected with miR-122 mimic showed lower cell viability than those transfected with NC, with significant difference detected at 48 and 72 h post-transfection (P<0.05 or P<0.01, Figure 2), whereas miR-122 inhibitor significantly promoted cell viability at 24, 48 and 72 h post-transfection (P<0.05). These results pointed out that miR-122 might decrease cell viability of mouse cardiomyocytes.

**Figure 3:**
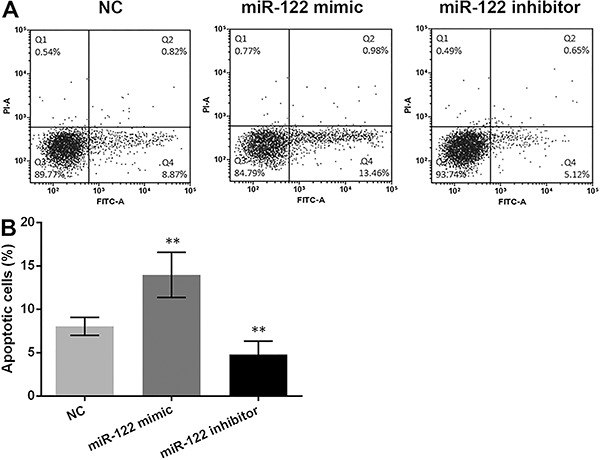
Apoptosis of transfected mouse cardiomyocytes. Mouse cardiomyocytes were transfected with miR-122 mimic, miR-122 inhibitor or the negative control (NC), and flow cytometry was performed at 48-h post-transfection. *A*, Flow cytometry results after the cells were treated with fluorescein isothiocyanate (FITC) and propidium iodide (PI). The FITC-positive and PI-negative cells (in the lower right quadrant) were considered apoptotic cells. *B*, Percent of apoptotic cells according to the flow cytometry results. Data are reported as means±SD. **P<0.01 compared to NC group (*t*-test).

To explore the effect of miR-122 on apoptosis of cardiomyocytes, cell apoptosis was analyzed by flow cytometry. It was denoted that miR-122 overexpression remarkably induced cell apoptosis (P<0.01, Figure 3A and B), while miR-122 down-regulation significantly reduced cell apoptosis compared with NC (P<0.01), indicating that miR-122 might play an important role in inducing cardiomyocyte apoptosis.

### MiR-122 regulated expression of caspase-8

**Figure 4:**
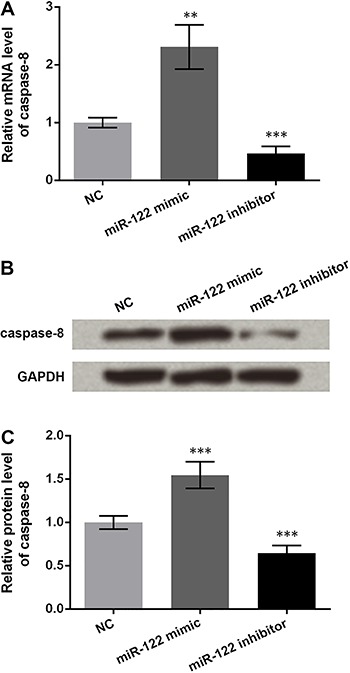
Expression of caspase-8 in transfected mouse cardiomyocytes. Mouse cardiomyocytes were transfected with miR-122 mimic, miR-122 inhibitor or the negative control (NC). qRT-PCR and western blot were performed at 48-h post-transfection to detect the mRNA and protein levels of caspase-8, respectively. *A*, mRNA level of caspase-8 detected by qRT-PCR. *B*, Western blot results showing the protein bands of caspase-8 and the internal reference GAPDH. *C*, Quantification of caspase-8 protein levels based on western blot results. Data are reported as means±SD. **P<0.01; ***P<0.001 compared to NC group (*t*-test).

To study the mechanism underlying the regulation of cardiomyocyte apoptosis by miR-122, the expression of caspase-8, which has been demonstrated to be the first step of the apoptosis executor caspase cascade (17), was analyzed. qRT-PCT showed that the mRNA level of caspase-8 was dramatically higher in cardiomyocytes transfected with miR-122 mimic (P<0.01, Figure 4A), and was obviously down-regulated in those transfected with miR-122 inhibitor, compared to NC (P<0.001). Western blot also showed the consistent changing pattern in caspases-8 protein levels (Figure 4B), and significant differences were detected in the quantified western blot results (P<0.001, Figure 4C). These findings suggest that miR-122 could regulate the expression of caspase-8.

## Discussion

Accumulating evidence has indicated that cardiomyocyte death is affected by apoptosis after myocardial infarction (4). This study found that miR-122 overexpression suppressed viability and promoted apoptosis of primarily cultured mouse cardiomyocytes, and miR-122 inhibitor had distinct effects. miR-122 also seemed to promote caspases-8 expression in cardiomyocytes.

Both miR-122 mimic and inhibitor caused significant changes in the miR-122 level, which led to profound alteration in cardiomyocyte viability and apoptosis. Previous findings have shown that miR-122 reduced viability but elevated apoptosis of hepatocellular carcinoma cells Huh-7 (18). Suppression of cell viability by miR-122 has also been detected in HepG2 and other tumor cells (19,20). MiR-122 enhances breast cancer cell apoptosis in combination with trastuzumab (21), and participates in germ cell apoptosis induced by ochratoxin A (22). Furthermore, tumor necrosis factor (TNF)-related apoptosis-inducing ligand expressing recombinant adenovirus regulated by miRNA response elements of miR-122 has shown an obvious advantage in accelerating apoptosis of breast cancer and esophageal cancer (23,24). In line with the above-mentioned evidence, our results also suggest the pro-apoptotic role of miR-122 in cardiomyocytes, and the opposite effects of miR-122 inhibitor.

MiR-122 has been reported to regulate various apoptotic factors in cell apoptosis. For example, B-cell CLL/lymphoma 2 (BCL2) and BCL extra-long were down-regulated, while p53 was increased by miR-122 in hepatoblastoma cells (25). This study analyzed the expression of caspase-8, an important apoptotic factor rapidly promoting the caspase cascade in response to apoptotic signals (26). Quite a few studies have reported the role of caspase-8 in the apoptotic mechanism of various cell types, such as melanoma A375 cells, neutrophils and head and neck carcinoma cells (27–29). In this respect, the up-regulation of caspase-8 by miR-122 found in this study was in concordance with the apoptotic role of caspase-8. Moreover, caspase-8 can be activated by miR-122 in hepatoma carcinoma cells BEL-7402/5-FU (30), thus we inferred that caspase-8 may be related to the mechanism of miR-122 in cardiomyocyte apoptosis.

The relationship between miR-122 and caspase-8 is intriguing. No evidence has been found to support the direct binding of caspase-8 mRNA by miR-122, thus it is reasonable to speculate that miR-122 influences the expression of caspase-8 via the mediation of other factors. Yu et al. (30), have indicated that the activation of caspase-8 can be promoted by miR-122, while the total caspase-8 level is suppressed in hepatocarcinoma cells. Our data were partly consistent with this previous study, both the active caspase-8 and total caspase-8 were up-regulated by miR-122 in cardiomyocytes. We speculate that miR-122 may regulate the transcription or translation of caspase-8 via other factors in cardiomyocytes. Several potential regulators of caspase-8 have already been investigated in previous research, such as Fas cell surface death receptor (31), Fas-associated via death domain (32) and TNF (33). Collectively, these reports suggest that a more extensive investigation into the integrated mechanism of miR-122 in cardiomyocytes is needed.

In summary, this study uncovered the promotive role of miR-122 in mouse cardiomyocyte apoptosis. The pro-apoptotic function of miR-122 may be related to the regulation of caspase-8. With further detailed elucidation of mechanisms, miR-122 can be a promising molecular target for the therapeutics of myocardial infarction.
